# Mediation effects of medication information processing and adherence on association between health literacy and quality of life

**DOI:** 10.1186/s12913-017-2598-0

**Published:** 2017-09-16

**Authors:** Sunmi Song, Seung-Mi Lee, Sunmee Jang, Yoon Jin Lee, Na-Hyun Kim, Hye-Ryoung Sohn, Dong-Churl Suh

**Affiliations:** 1 College of Pharmacy, Chung-Ang University, 84 Heukseok-ro, Dongjak-gu, Seoul, 06974 South Korea; 2 College of Pharmacy, Gacheon University, Incheon, South Korea

**Keywords:** Health literacy, Reading drug label, Understanding prescription instruction, Drug information seeking, Quality of life

## Abstract

**Background:**

To examine whether medication related information processing defined as reading of over-the-counter drug labels, understanding prescription instructions, and information seeking—and medication adherence account for the association between health literacy and quality of life, and whether these associations may be moderated by age and gender.

**Methods:**

A sample of 305 adults in South Korea was recruited through a proportional quota sampling to take part in a cross-sectional survey on health literacy, medication-related information processing, medication adherence, and quality of life. Descriptive statistics and structural equation modeling (SEM) were performed.

**Results:**

Two mediation pathways linking health literacy with quality of life were found. First, health literacy was positively associated with reading drug labels, which was subsequently linked to medication adherence and quality of life. Second, health literacy was positively associated with accurate understanding of prescription instructions, which was associated with quality of life. Age moderation was found, as the mediation by reading drug labels was significant only among young adults whereas the mediation by understanding of medication instruction was only among older adults.

**Conclusion:**

Reading drug labels and understanding prescription instructions explained the pathways by which health literacy affects medication adherence and quality of life. The results suggest that training skills for processing medication information can be effective to enhance the health of those with limited health literacy.

## Background

Health literacy, defined as the “degree to which individuals have the capacity to obtain, process, and understand basic health information and services needed to make appropriate health decisions,” is influenced by both social environments and characteristics of healthcare settings [1]. There is abundant evidence linking inadequate health literacy to a wide variety of negative health outcomes including increased hospitalizations, greater use of emergency care, inappropriate medication usage, poorer health status, and mortality [2]. Among various health management behaviors that are affected by health literacy, medication usage and adherence has received great empirical attention because of its direct impact on disease severity, quality of life, and increased healthcare costs [3, 4].

A meta-analysis has demonstrated inconsistent findings for the effects of health literacy on medication adherence [4]. Although the majority of previous studies exhibited a link between adequate health literacy and better medication adherence among adults with diabetes, human immunodeficiency virus infection, and heart failure [5–7], some studies refuted those findings and reported that low health literacy was associated with better medication adherence [8] or had non-significant associations [9]. These inconsistent findings suggest that cognitive and behavioral processes underlying the association between health literacy and medication adherence may involve complex causal pathways, which may not have been articulated in previous studies’ attempts to demonstrate a direct association between them.

By facilitating adherence to treatment regimen and self-management of a disease, health literacy has shown to ultimately contribute to an improvement in health related quality of life - an integral indicator for well-being and physical, psychological, and social function that are assessed from the patient’s view point [10]. A strong association between medication adherence and improved quality of life has been demonstrated in adults with chronic conditions [3, 11]. Although relatively few studies have examined the impact of health literacy on quality of life, the findings from those studies were mixed: some reported a significant impact of health literacy on quality of life [12], while others reported null findings [13]. Overall, the previous studies suggested that the processes by which health literacy affects medication adherence and quality of life need to be clarified based on the mixed findings on the associations among health literacy, medication adherence, and quality of life.

There are several theoretical models that identify specific processes by which health literacy may impact patient medication management and health-related quality of life [14–16]. These models suggest that low health literacy may put individuals at risk of having insufficient skills for acquiring and understanding medication-related information. Previous studies have provided limited support for these theoretical predictions such that adults with chronic disease and low health literacy often experience difficulties in understanding prescription drug labels or other health information [17, 18] and possess poor health information seeking skills [19]. These insufficient information acquisition and processing skills among those with low health literacy can lead to poorer understanding of prescribed medication regimens and medication non-adherence, which can further result in suboptimal management of disease symptoms and impaired quality of life.

This study aimed to investigate the mediating pathways by which medication-related information processing behavior and medication adherence explain the association between health literacy and health-related quality of life in South Korean adults. Firstly, the mediation pathways between health literacy and information processing tested whether lower levels of health literacy are associated with less active medication-related information processing behavior, including less reading of over-the-counter (OTC) drug labels; less accurate understanding of prescription instructions; and less seeking out of drug related information. Secondly, the medication pathways between information processing, medication adherence, and quality of life evaluated whether the indicators for less active medication-related information processing behavior led to poorer adherence to medication instructions and worsened quality of life. Additional exploration was conducted to test whether age and gender had moderating effects on mediating pathways, given the known influences of age and gender on health outcomes and health care utilization [20].

## Methods

### Model development

Based on previous literature and by adapting components from the integrative model, the causal pathway model, and the logic model of health literacy and health outcomes [14–16], we developed a theoretical model that explains mechanisms underlying the associations among health literacy, medication-related information processing, and quality of life. Our theoretical model predicts that health literacy may facilitate active medication-related information processing [14, 16] that can be indicated by thorough reading for drug labels, accurate interpretation of medication instructions, and frequent medication information seeking behaviors. As a result of active medication information processing, individuals may, in turn, become more likely to properly adhere to medication instruction, which can lead to better quality of life. Additionally, the model assumes that demographic variables such as education, age, gender, and other factors such as having a chronic disease may affect quality of life or directly or indirectly by influencing health literacy levels, medication-related information processing ability, or medication adherence [14, 15]. Chronic diseases which were included in the model included hypertension, cerebrovascular stroke, myocardial infarction (angina), hyperlipidemia, osteoarthritis, rheumatoid arthritis, tuberculosis, thyroid diseases, stomach cancer, liver cancer, colorectal cancer, breast cancer, cervical cancer, lung cancer, thyroid cancer, depression, atopic dermatitis, kidney failure, hepatitis B, hepatitis C, and liver cirrhosis.

### Study participants

Study participants were selected to have a nationally representative sample of the Korean adult non-institutionalized population from the provinces of Seoul and Gyeong-gi using a proportional quota sampling method, stratified by age and gender. Almost half (46%) of the total population of South Korea resides in these two provinces. Exclusionary criteria included participation in other national surveys within the past 6 months or current employment in the healthcare, research, or marketing industries. A total of 305 participants completed a cross-sectional survey which assessed health literacy, medication usage, health status, and demographic information; 50.5% of these participants were female and the mean age was 41.76 ± 13.15 years.

### Data collection procedures

All survey data were collected via multiple group survey sessions conducted in October–December, 2014 that were organized by trained survey moderators and interviewers. Multiple group survey sessions were conducted with up to 30 participants per session. During a group survey session, a survey moderator would introduce the survey and answer any questions about the survey from participants. All participants completed and signed an informed consent form before the survey started. This study was approved by the Institutional Review Board of Chung-Ang University (approval number: 1,041,078–201,409-HR-136-01).

### Measures

Health Literacy: Health literacy was assessed using the translated Korean version of the Rapid Estimate of Adult Literacy in Medicine (REALM), which is a word recognition test of common medical words and layman terms relating to body parts and illnesses [21]. The Korean version of the REALM was developed by incorporating culturally appropriate translations for the 66 words from the REALM and modifying its administration and rating [22]. Instead of reading each of the 66 words aloud, participants responded to a written questionnaire that asked whether they knew each of the words on the list using a 4-point Likert scale (1 = I don’t know this term, 2 = I have seen the term before but don’t know the meaning, 3 = I have seen the term before and know its meaning a little, 4 = I know this term). These 4-point scale values were reclassified to either 0 which equated to not knowing the word (including the 1 and 2 responses) or 1 which equated to knowing the word (including the 3 and 4 responses). Consequently, the total score range was 0–66.

The cut-off score of 61 on the REALM was then used to classify participants as having adequate versus inadequate health literacy, which has been validated by previous studies with Korean adolescent and adult samples [22, 23]. The internal consistency of the scale for this sample was α = 0.96.

Reading labels of non-pharmacy sales of OTC drugs: Participants’ level of label reading of non-pharmacy sales of OTC drugs was assessed based on how many of the listed six information components were usually read from each of three selected OTC drugs (cold medicines, nonsteroidal anti-inflammatory agents for children, and digestive medications). The study’s OTC drugs were sold at non-pharmacy locations (e.g., convenience stores, supermarkets) in order to increase general access to OTC drugs when pharmacies were closed. The sample drug labels were taken from actual labels of available OTC medications, and the six subsections included active ingredient(s), product type, uses, dosage, warnings, and cautions. Participants rated their levels of reading of each sample label subsection using a 5 Likert point scale, ranging from 1 = ‘not read any label information’ to 5= ‘reading all information within the label section’. The internal consistency of the scale was α = 0.96.

Understanding prescription instructions: Participants’ understanding of prescription instructions was measured based on 5 multiple choice questions for each of four different prescriptions, resulting in a total of 20 questions. These questions were designed to test whether participants can accurately understand four different prescriptions. The instructions associated with each prescription included a list of all the prescribed medicines for a patient; directions on how to take the medicine; the uses; and dosage. The 20 questions about the prescription instructions asked, for example, “According to the prescription instructions, how many prescription medications does the patient need to take at lunch time?” A total score was calculated by counting the number of correct answers, ranging from 0 to 20.

Medication-related information seeking behavior: To assess medication-related information seeking behavior, participants were asked how frequently they use different sources of information including asking healthcare professionals, using online resources, and reading printed materials. The frequency of using each source was rated on a 5-point Likert scale from 1 = ‘not using it at all’ to 5 = ‘very frequently using it’.

Medication adherence: Medication adherence was assessed based on responses to the following question that was adapted from the Korea Health Panel Survey [24]: ‘When you take your medication, how likely are you to follow the instructions of how to take your medication?’ with a 5-point Likert scale ranging from 1 = ‘not very likely to follow the instructions’ to 5 = ‘very likely to follow the instructions’. This question measures self-perception of the general tendency to follow medication instructions rather than asking whether a person took specific medications as instructed. Respondents’ perception of their medication adherence as a proxy for actual medication taking behaviors shows high correlation with other non-self-reported medication adherence records [25].

Quality of life: Participants’ quality of life was measured using the Korean version of the Short Form-36 health survey, which was validated for its reliability and its ability to detect health status and health-related quality of life among those with and without disease conditions across different countries [26]. The scale values of Short Form-36 health survey were used to derive a health utility value, which is a preference-based single index measure of health-related quality of life based on the SF-6D algorithm [27]. The SF-6D is composed of the ordinal levels of health states in six dimensions including physical functioning, role limitations due to emotional and physical health problems, social functioning, pain, mental health, and vitality. The six dimensions were used to calculate preference weighted health utility scores that ranged from 0 to 1.

### Statistical analysis

The demographic characteristics and levels of key study variables between people with adequate and inadequate health literacy were compared using the Student t-test for continuous variables with a normal distribution, the Mann-Whitney U test for variables without a normal distribution, and the Chi-square test for categorical variables.

After the correlations of key study variables were examined, structural equation modeling (SEM) was conducted for testing the associations between health literacy, medication-related information processing, perceived medication adherence, and quality of life using AMOS version 23.0 (SPSS Inc., Chicago, IL). First, latent variables were defined using factor analysis to identify groups of items that assess the different aspects of a construct (e.g., reading OTC drug labels and seeking out medication-related information). Second, the direct pathways between health literacy and quality of life and the mediating pathways through medication-related information processing and perceived medication adherence were added to the model to estimate the mediation effects of medication-related information processing. The mediation effect was then tested by the joint significance test [28]. This test examines the significance of coefficients of both α pathways (i.e., the pathways between predictor and mediator variables) and β pathways (i.e., the pathways between mediator and outcome variables) to determine the mediation effects. Demographic and clinical variables such as age, gender, education, and chronic diseases were initially connected to study variables, but those pathways from the demographic variables were removed in the final SEM model as they were not significantly associated with the study variables.

Third, bootstrapping analyses were conducted with 1000 bootstrapped samples in order to obtain reliable 95% confidence intervals of estimated parameters which were not normally distributed. The model fit indices, including root mean-squared error of approximation, Tucker-Lewis index, and the comparative fit index were calculated, and the cutoff scores of root mean-squared error of approximation <0.10 and Tucker-Lewis index and comparative fit index >0.90 were used to evaluate the model [45, 46]. Additionally, the chi-square statistic for the model fit was obtained by applying the bootstrapped sampling distribution and Bollen-Stine statistics [29].

Finally, gender and age moderation effects for the mediational pathways were examined using a multi-group structural equation modeling technique. After removing gender (or age) from the model, all of the paths of the final model were constrained to be equal for women and men (or young adults 20–39 years old and older adults 40–69 years old). Then, the model was then compared to an otherwise identical model in which all paths were not constrained to be equal between women and men or between young and old adults. A chi-square difference test (Δ *χ* ^*2*^) was then used to determine if the fit of the constrained model was significantly worse than the one without constraints [30].

## Results

As presented in Table 1, participants were highly educated, and 30.5% of them had at least one chronic disease conditions such as hypertension, arthritis, or diabetes. Inadequate health literacy was less prevalent among middle-aged adults than their younger or older counterparts. Compared to those with adequate health literacy, those with inadequate health literacy tended to be less educated, have one or more chronic diseases, and reported lower perceived medication adherence but similar levels of quality of life. Table 2 shows correlations between pairs of study and demographic variables. Health literacy was positively correlated with all three indicators for medication-related information processing, but not with quality of life. Quality of life was positively associated with perceived medication adherence, understanding prescription instructions, and education but was negatively associated with age, gender, and chronic disease.

**Table 1:** Demographic characteristics of participants by health literacy level

	All participants (*n* = 305) n (%)	Adequate health literacy (*n* = 198) n (%)	Inadequate health literacy (*n* = 107) n (%)	*p*-value
Gender				
Women	154 (50.49)	102 (51.52)	52 (51.40)	0.63
Men	151 (49.51)	96 (48.48)	55 (48.60)	
Age (mean ± SD)	(41.76 ± 13.16)	(43.06 ± 12.24)	(39.36 ± 14.46)	< 0.05
20–34	100 (32.79)	56 (28.28)	44 (41.12)	< 0.05^a^
35–54	147 (48.20)	105 (53.03)	42 (39.25)	
55–59	21 (6.89)	16 (8.08)	5 (4.67)	
≤ 60	37 (12.13)	21 (10.61)	16 (14.95)	
Education				
< High school	48 (15.74)	25 (12.63)	23 (21.50)	< 0.001^a^
Less than college	44 (14.43)	18 (9.09)	26 (24.30)	
College	196 (64.26)	141 (71.21)	55 (51.40)	
Graduate School	17 (5.57)	14 (7.07)	3 (2.80)	
Number of chronic disease^b^				
None	212 (69.51)	135 (68.18)	77 (71.96)	0.49
≥ 1	93 (30.49)	63 (31.82)	30 (28.04)	
Medication adherence^c^, mean ± SD	3.96 ± 0.81	4.05 ± 0.72	3.79 ± 0.95	< 0.05
Quality of life^d^, mean ± SD	0.74 ± 0.12	0.74 ± 0.12	0.74 ± 0.12	0.76

Abbreviation: *n*: number *SD* standard deviation

^a^ Fisher’s exact test was used because the expected cell size ≤5. ^b^ Chronic disease is defined as a disease (such as hypertension, arthritis, diabetes, and asthma, etc) that persists for 3 months or longer. ^c^ Scores range from 1 to 5. Higher scores reflect better medication adherence. ^d^ Scores range from 0 to 1. Higher scores represent better health related quality of life

**Table 2:** Correlations among measured indicators

	1	2	3	4	5	6	7	8	9	10
1. Quality of life	1	0.14*	0.07	−0.06	0.29**	−0.08	−0.26**	−0.16**	0.16**	−0.15**
2. Medication adherence		1	0.04	0.25**	0.08	0.12*	0.12*	−0.15**	−0.004	0.12*
3. Health literacy			1	0.15**	0.18**	0.16**	−0.03	0.09	0.12*	0.07
4. Reading drug labels				1	−0.10	0.49**	0.29**	0.14*	0.004	0.11
5. Understanding prescriptions					1	−0.07	−0.49**	−0.12*	0.33**	−0.19**
6. Information seeking behavior						1	0.14*	0.08	0.08	0.06
7. Age							1	0.02	−0.17**	0.40**
8. Gender								1	−0.19**	−0.01
9. Education									1	−0.004
10. Chronic disease										1

* p < 0.05, ** p < 0.01

As presented in Table 3, the two measurement models for reading labels and information seeking behavior were confirmed to be appropriately established, with significant factor loadings connecting measured variables with a defined latent variable (factor loadings 0.46–0.94, all *p* < 0.01). The items for reading OTC drug labels were classified into three components including: a) reading cautions and warnings, b) reading use and dosage, and c) reading active ingredients and product type. In addition, the items assessing medication-related information seeking behavior were classified into a) searching for internet, b) asking healthcare professionals, and c) reading books and magazines according to the underlying factor structure (Table 4). When item mean scores by age groups were analyzed, older adults appeared to read more warnings/cautions and active ingredients/product type sections of drug labels (all *p* < 0.01) and used less internet sources and more printed materials when searching for medication-related information than young adults (all p < 0.01). The mean scores from each category of items were used to establish measurement models.

**Table 3:** Factor structure of over-the-counter drug label reading level by age group

Items	Factor loading	Total (*n* = 305) mean ± SD	Young adults (*n* = 141) mean ± SD	Older adults (*n* = 164) mean ± SD	*p*-value
Factor I. Reading cautions and warnings					
Cold medicine: The people with any of the following cases must not taking this medication	0.77	3.30 ± 1.31	3.00 ± 1.27	3.56 ± 1.28	< 0.001
Cold medicine: Do not take this medication with following medication	0.79	3.33 ± 1.38	2.89 ± 1.41	3.71 ± 1.23	< 0.001
Cold medicine: Ask a healthcare professional if you have any of the following health conditions	0.87	2.88 ± 1.35	2.47 ± 1.33	3.24 ± 1.27	< 0.001
Cold medicine: Stop taking this medication and consult with a healthcare professional if you experience any of the following symptoms	0.86	2.86 ± 1.34	2.46 ± 1.31	3.21 ± 1.27	< 0.001
Digestive medicine: Stop taking this medication and consult with a healthcare professional for following cases.	0.79	3.60 ± 1.19	3.33 ± 1.21	3.83 ± 1.12	< 0.001
Digestive medicine: Where to report if you experience any side effects	0.76	2.63 ± 1.48	2.08 ± 1.29	3.11 ± 1.46	< 0.001
Digestive medicine: Storage information	0.71	3.04 ± 1.42	2.66 ± 1.44	3.37 ± 1.32	< 0.001
Pediatric NSAID: The people with any of the following cases must not taking this medication	0.81	2.87 ± 1.44	3.13 ± 1.19	3.81 ± 1.13	< 0.001
Pediatric NSAID: Do not take this medication with other NSAIDs and avoid overdosing	0.86	3.40 ± 1.26	3.09 ± 1.26	3.68 ± 1.19	< 0.001
Pediatric NSAID: Ask a healthcare professional about taking this medication if you have any of the following conditions	0.90	3.05 ± 1.40	2.54 ± 1.34	3.48 ± 1.30	< 0.001
Pediatric NSAID: Stop taking this medication if you experience any of the following symptoms	0.85	2.92 ± 1.35	2.43 ± 1.28	3.35 ± 1.26	< 0.001
Pediatric NSAID: Warnings	0.81	2.96 ± 1.39	2.57 ± 1.39	3.29 ± 1.30	< 0.001
Pediatric NSAID: When taking this medication for cold symptoms, do not take this medication for more than 5 days	0.76	3.05 ± 1.43	2.67 ± 1.43	3.37 ± 1.35	< 0.001
Factor II. Reading uses and dosage					
Cold medicine label: Uses	0.68	4.30 ± 0.91	4.37 ± 0.83	4.24 ± 0.98	0.21
Cold medicine label: Dosage	0.68	4.55 ± 0.81	4.56 ± 0.83	4.55 ± 0.80	0.90
Digestive medicine label: Uses	0.78	4.38 ± 0.84	4.30 ± 0.96	4.34 ± 0.87	0.73
Digestive medicine label: Dosage	0.82	4.52 ± 0.78	4.40 ± 0.97	4.51 ± 0.74	0.24
Pediatric NSAID label: Uses	0.77	4.32 ± 0.91	4.32 ± 0.86	4.43 ± 0.82	0.26
Pediatric NSAID label: Dosage	0.69	4.46 ± 0.85	4.38 ± 0.93	4.63 ± 0.60	< 0.05
Factor III. Reading active ingredients and product type					
Digestive medicine: Active ingredients	0.81	2.17 ± 1.22	1.91 ± 1.11	2.38 ± 1.28	< 0.01
Digestive medicine: Product type	0.82	2.53 ± 1.41	2.28 ± 1.37	2.74 ± 1.42	< 0.01
Pediatric NSAID: Active ingredients	0.80	2.48 ± 1.35	2.28 ± 1.32	2.65 ± 1.36	< 0.05
Pediatric NSAID: Product type	0.83	2.70 ± 1.41	2.47 ± 1.38	2.90 ± 1.41	< 0.01

Abbreviations: *n* number, *SD* standard deviation, *NSAID* non-steroidal anti-inflammatory drug

Factor I: Reading cautions and warnings: Cronbach α = 0.96; eigenvalue = 11.43; total variance explained = 49.73%

Factor II: Reading dosage and uses: Cronbach α = 0.88; eigenvalue = 3.02, total variance explained = 13.14%

Factor III: Reading active ingredients and product type: Cronbach α = 0.89; eigenvalue = 1.42, total variance explained = 6.18%

**Table 4:** Factor structure of medication related information seeking behavior level by age group

Items	Factor loading	Total (n = 305) mean ± SD	Young adults (*n* = 141) mean ± SD	Older adults (*n* = 164) mean ± SD	*p*-value
Factor I. Asking healthcare professionals					
Asking a pharmacist	0.89	3.36 ± 1.07	3.29 ± 1.08	3.43 ± 1.06	0.27
Asking a doctor	0.86	2.80 ± 1.19	2.71 ± 1.20	2.88 ± 1.18	0.20
Asking a nurse	0.77	2.35 ± 1.09	2.25 ± 1.10	2.43 ± 1.08	0.14
Factor II. Using the internet					
Using general search engines	0.80	3.34 ± 1.16	3.66 ± 1.03	3.07 ± 1.20	< 0.001
Using governmental websites	0.78	1.88 ± 0.94	1.84 ± 1.03	1.92 ± 0.87	0.45
Using pharmaceutical companies’ websites	0.76	1.93 ± 0.97	1.77 ± 0.94	2.07 ± 0.98	< 0.01
Factor III. Using printed materials					
Reading health-related magazines	−0.86	1.44 ± 0.70	1.24 ± 0.51	1.60 ± 0.80	< 0.001
Reading books on health topics	−0.85	1.71 ± 0.93	1.45 ± 0.77	1.95 ± 1.00	< 0.001

Abbreviations: *n* number, *SD* standard deviation

Factor I: Asking healthcare professionals: Cronbach α = 0.79; eigenvalue = 2.98; total variance explained = 37.28%

Factor II: Using the internet: Cronbach α = 0.68; eigenvalue = 1.62; total variance explained = 20.29%

Factor III: Using printed materials: Cronbach α = 0.72; eigenvalue = 1.10; total variance explained = 13.76%

**Fig. 1:**
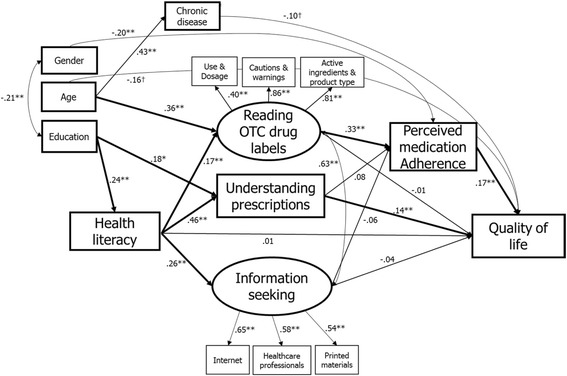
Model of the hypothesized mediating pathways with standardized regression coefficients. * *p* < 0.05, ** *p* < 0.01, † 0.05 < *p* < 0.10. For all variables, higher values indicate higher levels of construct. The latent constructs are represented as ovals and the measured variables as rectangles. Key variables are shown in boldface

Figure 1 presented the final SEM model which has two significant mediation pathways. The first mediation pathway showed the associations among health literacy, reading drug labels, perceived medication adherence, and quality of life. Particularly, higher health literacy was associated with more thorough reading of drug labels (B = 0.17; 95% CI = 0.07–0.29), which was associated with better perceived medication adherence (B = 0.33; 95% CI = 0.15–0.49). The perceived medication adherence was, in turn, associated with higher quality of life (B = 0.17; 95% CI = 0.08–0.28). The other mediation pathways were comprised of associations between health literacy, understanding prescription instructions, and quality of life. In the second mediation pathway, higher health literacy was associated with more accurate understanding of prescription instructions (B = 0.46; 95% CI = 0.32–0.62). More accurate understanding was, in turn, linked with higher quality of life (B = 0.14; 95% CI = 0.04–0.26). Health literacy was positively associated with medication-related information seeking behavior (B = 0.26; 95% CI = 0.10–0.42), which was not associated with both perceived medication adherence (B = −0.06; 95% CI = −0.20 – 0.13) and quality of life (B = −0.04; 95% CI = −0.21 – 0.17). The model fit was good with χ^2^ (54) = 97.19, *p* < 0.01, Tucker-Lewis index = 0.92, comparative fit index = 0.95, root mean-squared error of approximation = 0.05 (90% CI = 0.03–0.07).

**Fig. 2:**
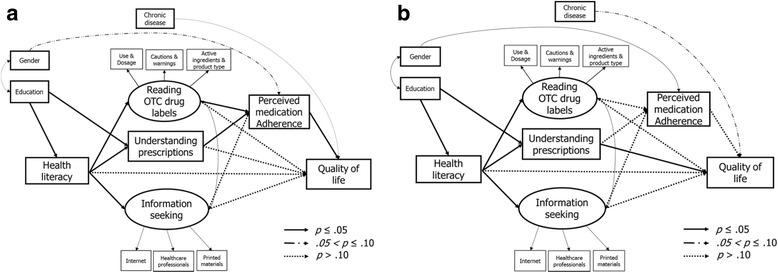
Pathways of the model stratified by age (**a**) young adult (*n* = 141) and (**b**) older adult group (*n* = 164). The various arrows can be interpreted as follows: the solid arrow indicates a pathway being significant at *p* ≤ 0.05, the dashed arrow at 0.05 < *p* ≤ 0.10, and the dotted arrow at *p* > 0.10

As presented in Fig. 2 (a) and (b), the multi-group SEM found the age moderation effects on the pathways in the final model with the significant chi-square difference test result (Δχ^2^(15) = 89.98, *p* < 0.01). Although health literacy was associated with more reading of the OTC drug labels in both age groups (B = 0.18, *p* ≤ 0.05 for young adults, B = 0.18, p ≤ 0.05 for old adults), reading the OTC drug labels was linked to perceived medication adherence (B = 0.37, p < 0.01), which was associated with quality of life (B = 0.25, p < 0.01) only in the young adult group. On the other hand, the mediation effect of understanding medication information was significant only among old adults: higher levels of health literacy were associated with better understanding of medication instructions (B = 0.35, p < 0.01), which was, in turn, associated with improved quality of life (B = 0.26, p < 0.01) among old adults.. Gender moderation was not statistically significant.

## Discussion

The present study tested a theoretically-driven SEM model that suggested that efficient medication-related information processing and medication adherence can be the mechanisms understanding poor health literacy and health problems among Korean adults. The current results suggest that poor health literacy may lead to worse quality of life by inducing inefficient medication-related information processing and poor medication adherence, which was demonstrated by the two significant mediational pathways. The first mediation pathway was that lower health literacy was associated with less reading of OTC drug labels, which was linked to lower levels of quality of life by inducing lower perceived medication adherence. The other mediation pathway showed that lower health literacy was linked to a less accurate understanding of prescription instructions, which was in turn was associated with a higher quality of life. Finally, the present study also suggests that age may influence the magnitude of mediation of reading drug labels such this the mediation by reading labels was significant only in young adults.

The present results showed that health literacy is positively associated with all three indicators of medication-related information processing including reading drug labels, understanding prescription instructions, and information seeking behavior. The results are consistent with previous literature which demonstrated the broad influence of health literacy on knowledge, interpretation, and a search for health information among adults with a chronic disease condition [12, 31, 32]. For example, low health literacy was associated with the inability to read all contents on prescription drug labels in seniors and patients at a primary care clinic [17, 33], misinterpretation of a prescription medicine label in adults with human immunodeficiency virus infection and patients at a primary care clinic [17, 31], and utilization of various sources to seek out health information in people with diabetes [19]. Overall, our results replicate previous findings, showing that inadequate health literacy can be the common underlying factor in explaining difficulty acquiring and comprehending medication-related information among adults with and without a chronic condition.

Among the three indicators for medication-related information processing, reading drug labels mediated the associations between health literacy and medication adherence. The results revealed a cascade of effects that initiated from lower health literacy, resulting in less reading for OTC drug labels, which was, in turn, connected to lower medication adherence and worse quality of life. These findings suggest that less reading for drug labels may put people at greater risk for unintentional misuse of OTC drugs resulting from a lack of knowledge about proper use. Medication misuse and poor adherence have been consistently recognized as causal factors influencing the process by which low health literacy leads to adverse health outcomes [34, 35]. These results imply that healthcare professionals should be aware that individuals with limited health literacy are not likely to read all information on their medication instructions, which may have negative consequences on their health, including medication non-adherence and impaired quality of life.

In addition to reading drug labels, the results indicate that understanding prescription instructions may explain how low health literacy can adversely impact quality of life. Previous studies have reported that low health literacy is associated with misunderstanding of dosage, warnings, and other important information about prescribed medicine among adults [17, 36]. Inaccurate understanding of prescription instructions is known to put people at risk for unintended misuse of prescribed medicines and subsequent hospital admissions because of medication misuse [37]. Therefore, the results suggest that interventions to better interpret prescription instructions can be an effective way to help people with low health literacy improve their health and quality of life.

Perhaps unexpectedly, the current results showed that medication-related information seeking behavior only marginally accounted for the associations among health literacy, medication adherence, and quality of life. This weak effect may potentially be attributed to the varying qualities of health information that is available on the internet [38] and from other sources. Future study is needed to verify whether individuals find both misleading and accurate health information during information seeking behavior and experience difficulties in filtering out accurate versus inaccurate health information.

Interestingly, this study found differences between younger and older adults in the examined pathways. The reading of OTC drug labels accounted for the association between health literacy and perceived medication adherence only in young adults. Previous studies have shown that adolescents and young adults are less likely to read OTC drug labels for common symptoms like headaches, [39] which may explain the important role that reading label information has in enhancing medication adherence among young adults. In contrast, as seen only among older adults, a more accurate understanding of medication instructions explained the process by which higher health literacy can lead to an improved quality of life. A recent study reached a conclusion that is consistent with the current results among older adults: in adults with hypertension, an age-related decline in the ability to process health information that is critical for self-care was compensated for by their knowledge of health literacy [40, 41]. Overall, the age moderation results indicate that young adults and older adults may have different applications in their use of medication-related health information.

This study is the first to demonstrate that the theoretically driven mediational pathways explain how poor health literacy can be translated into less medication adherence and lower quality of life via inefficient medication-related information processing. In addition, this study extends the previous findings, which focused on examining impacts of health literacy on health outcomes among adults with a chronic disease, by involving adults both with chronic disease conditions and those that were healthy from ages 20 to 69. However, several limitations should be considered when interpreting this study’s results. First, the majority of participants were highly educated adults living in an urban area in South Korea. Thus, this study results may be generalizable to other populations with caution. Second, the current data were collected by using self-reported questionnaires and might have been influenced by response bias and recall errors of participants [42]. Third, our study sample did not include older adults over 70 years old because our study design was unable to accommodate older adults with limited mobility. Finally, the study design was cross-sectional survey with a limited sample size and could not examine causal relationships.

The present results provide the rationale for developing cognitive skill-based interventions for adults with limited health literacy [16] and describe the specific role of particular skills in acquiring and understanding medication-related information. This study results suggest that developing skills for thorough reading and accurate understanding of OTC drug labels and prescription instructions may improve medication adherence and quality of life among adults with limited health literacy. Particularly, young adults with limited health literacy may need to be advised to completely read labels on OTC drugs, and trained in aspects of efficient information seeking behavior to contribute to better health outcomes for older adults. Overall, the findings suggest the potential benefits of educational interventions that focus on developing skills to effectively read, understand, and apply health information for managing medication regimens in enhancing quality of life of adults with limited health literacy.

## Conclusions

The current study demonstrated the importance of medication-related information processing in explaining the pathways by which limited health literacy impairs medication adherence and health related quality of life. Lower health literacy appears to be associated with poorer medication adherence and lower quality of life via reading less information on a drug label or not accurately understanding prescription instructions, but the magnitudes of these associations might differ between younger and older adults.

## Abbreviations

CI: Confidence interval
OTC: Over-the-counter
REALM: Rapid estimate of adult literacy in medicine scale
SD: Standard deviation
SEM: Structural equation modeling
